# Effect of growth media on the MTT colorimetric assay in bacteria

**DOI:** 10.1371/journal.pone.0219713

**Published:** 2019-08-27

**Authors:** Ludmil Benov

**Affiliations:** Department of Biochemistry, Faculty of Medicine, Kuwait University, Kuwait City, Kuwait; National Research Council, ITALY

## Abstract

Reduction of tetrazolium salts to colored formazan products by metabolically active cells is widely used for assessment of cell viability. Among the tetrazolium compounds most commonly used is MTT [3-(4,5-dimethylthiazol-2-yl)-2,5-diphenyltetrazolium bromide]. Numerous studies about sites and mechanisms of cellular reduction of MTT, performed in mammalian cell cultures, have identified various parameters that affect formazan production and can lead to overestimation/underestimation of viable cells or effects of treatment. Irrespective of lack of such data for prokaryotic cells, the MTT assay is commonly used for microbiological studies, which often leads to contradictory results or misinterpretation of data. The aim of this study was to investigate how components of growth media and conditions of growth, affect formazan formation by microbial cells. Results showed that MTT reduction depended on the amino acid composition of the medium. Several amino acids potentiated formazan production by Gram-positive and Gram-negative bacteria, with histidine having the strongest effect. Results of this study demonstrate that data obtained with the MTT test should be interpreted with caution, particularly when different growth media are used or treatments affect metabolic pathways, and that evaluation of the reliability of the MTT assay under specific conditions should be performed, to avoid erroneous results. Performing the assay with cells suspend in glucose-supplemented buffer would eliminate the effects of metabolites and will limit cell division during incubation with MTT. Another critical element to be considered is the choice of a proper solvent for dissolution of formazan crystals.

## Introduction

Assessment of microbial responses to treatments and microbial viability in general, is commonly based on ability to reproduce. Preparations of cell suspensions and enumeration of colonies, however, is time and labor demanding. In addition, cell responses to treatment are not always limited to ability to replicate, and inability to reproduce does not always mean loss of viability [1–4]. Inconveniences and limitations of traditional microbiological methods prompted interest in adopting new techniques. Colorimetric procedures are economical, rapid, can measure multiple samples simultaneously, can be automated, and are preferred for evaluation of the physiological state of microbes. Most often, such colorimetric tests are based on enzymatic reduction of colorless or light colored tetrazolium salts to strongly colored formazan products [5]. Among the tetrazolium salts most frequently used for bioassays is 3-(4,5-Dimethylthiazol-2-yl)-2,5-diphenyltetrazolium bromide (MTT) [6, 7]. MTT-based assays have been widely used to evaluate microbial physiological state, including assessment of viability and growth.

While numerous publications deal with the mechanism of reduction of MTT to formazan by mammalian cells (for a detailed review see [8]), investigations on the mechanism of reduction of MTT by microbial cells are rather limited (reviewed in [6]). MTT protocols adopted by different laboratories vary wildly with respect to assay conditions [9, 10], which often leads to misleading results and misinterpretations. Parameters that affect MTT reduction by mammalian cells have been extensively investigated [10], but very few works investigated variables that affect the ability of microbial cells to reduce MTT [6, 11, 12].

The aim of this study was to investigate how media composition and conditions of growth, affect reduction of MTT to formazan by Gram negative and Gram-positive bacterial species, *E*. *coli* and *S*. *aureus*.

## Materials and methods

### Strains and growth conditions

The following Gram-negative and Gram-positive strains were used in this study: *Escherichia coli* strain GC4468 (F^-^ Δlac U169 *rpsL*) provided by Dr. D. Touati [13], and *Staphylococcus aureus* strain ATCC25923 [14]. Overnight cultures were grown in a shaking water bath at 200 rpm and 37°C in Luria-Bertoni (LB) medium (BD Biosciences, USA) or in Brain Heart Infusion (BHI) broth (BD Biosciences, USA), respectively.

For experiments, the overnight cultures were either diluted 200-fold in LB medium and grown to mid-log phase in a shaking water bath at 200 rpm and 37°C or diluted and used as stationary phase cultures. When necessary, cells were thoroughly washed with PBS to remove traces of medium and resuspended to OD_600nm_ = 0.5 or 0.1 in PBS containing 0.2% glucose. Experiments were performed in 96-well plates. Aliquots (100 μl/well) of cell suspensions were transferred into triplicate wells, and tested compounds were added. Stock solutions of tested compounds were prepared in PBS and filter-sterilized.

M9CA medium consisted of M9 salts, prepared by dissolving 6 g Na_2_HPO_4_, 3 g K_2_HPO_4_, 1 g NH_4_Cl, and 0.5 g NaCl in 1 liter of distilled water. To 100 ml of M9 salts, 1 ml of each 0.2 M MgSO4, 20% glucose, casamino acids (Difco) to 20%, and 50 μL of 0.2 M CaCl_2_, autoclaved separately were added. Immediately before use, filter-sterilized solutions of pantothenic acid and thiamine were added to a final concentration of 3 mg/L.

Individual amino acids (Sigma-Aldrich) were added to phosphate-buffered saline (PBS) (Thermo Fisher Scientific) supplemented with 0.2% glucose or to M9 salts supplemented with 0.2% glucose. Since both media produced similar results, only data obtained in PBS/glucose are presented.

For anaerobic experiments, solutions were degassed, and together with plates containing cell suspensions, were transferred and equilibrated in an anaerobic Coy chamber (Plas Labs Inc., USA). All additions were performed under anaerobic conditions. Anaerobic cultures were grown in a Coy chamber at 37 ^o^C.

### MTT assay

MTT reagent was prepared by dissolving 25 mg of 3-(4,5-dimethylthiazol-2-yl)-2,5-diphenyl-tetrazolium bromide (MTT) (Sigma-Aldrich) in 5 ml PBS. Unless otherwise specified, 10% SDS in 10 mM HCl was used for solubilization of the formazan crystals. Other tested solubilization solvents were 10% SDS dissolved in 50 mM phosphate buffer, pH 7.4, dimethyl sulfoxide (DMSO), ammonia-containing DMSO [15], and isopropanol. To improve solubility, half of the wells containing identical samples were first treated with SDS dissolved in distilled water (final concentration in wells 5%) or solubilized with organic solvents containing 5% SDS.

Ten μl of MTT reagent were added to all wells and plates were incubated for 30 min on a thermostatic shaker at 37 ^o^C and 200 rpm in the dark. After 30 min, 100 μl of SDS HCl reagent, SDS in phosphate buffer, ammonia-DMSO, DMSO or isopropanol, with and without SDS were added per well and plates were incubated for 1 h at room temperature in the dark. In cases incomplete formazan dissolution was detected, the time of incubation was extended to 3 hours. The absorbance of each well at 560 nm and 700 nm was measured and absorption spectra of the wells were recorded using a microplate reader (CLARIOstar, BMG LABTECH Inc., USA). Data was analyzed using MARS Data Analysis software provided by the manufacturer.

In all experiments controls containing reagents but no cells were run in parallel.

### Cellular respiration

Cellular respiration was measured using Biological Oxygen Monitor System (YSI 5300A, YSI Inc., Yellow Springs, USA) containing a thermostatic chamber equipped with magnetic stirrer and fitted with a Clark electrode. The system has been connected to a two-channel chart recorder, and has been calibrated before each set of experiments according to the manufacturer’s instructions. Five hundred μl aliquots of cell suspensions (OD_600 nm_ = 0.5) were transferred into the electrode chamber filled with 2.5 ml of PBS supplemented with 0.2% glucose. Oxygen consumption was recorded and calculated as nmol O_2_ consumed/min/ml cell suspension. Blanks containing all reagents but no cells, were run in parallel.

### Data analysis

Each experiment was repeated at least two times with not less than three replicates. One Way Analysis of Variance (ANOVA) was performed using SigmaPlot version 11.0 and p value < 0.05 was accepted as statistically significant. Data is presented as mean ± SD.

## Results and discussion

**Fig 1:**
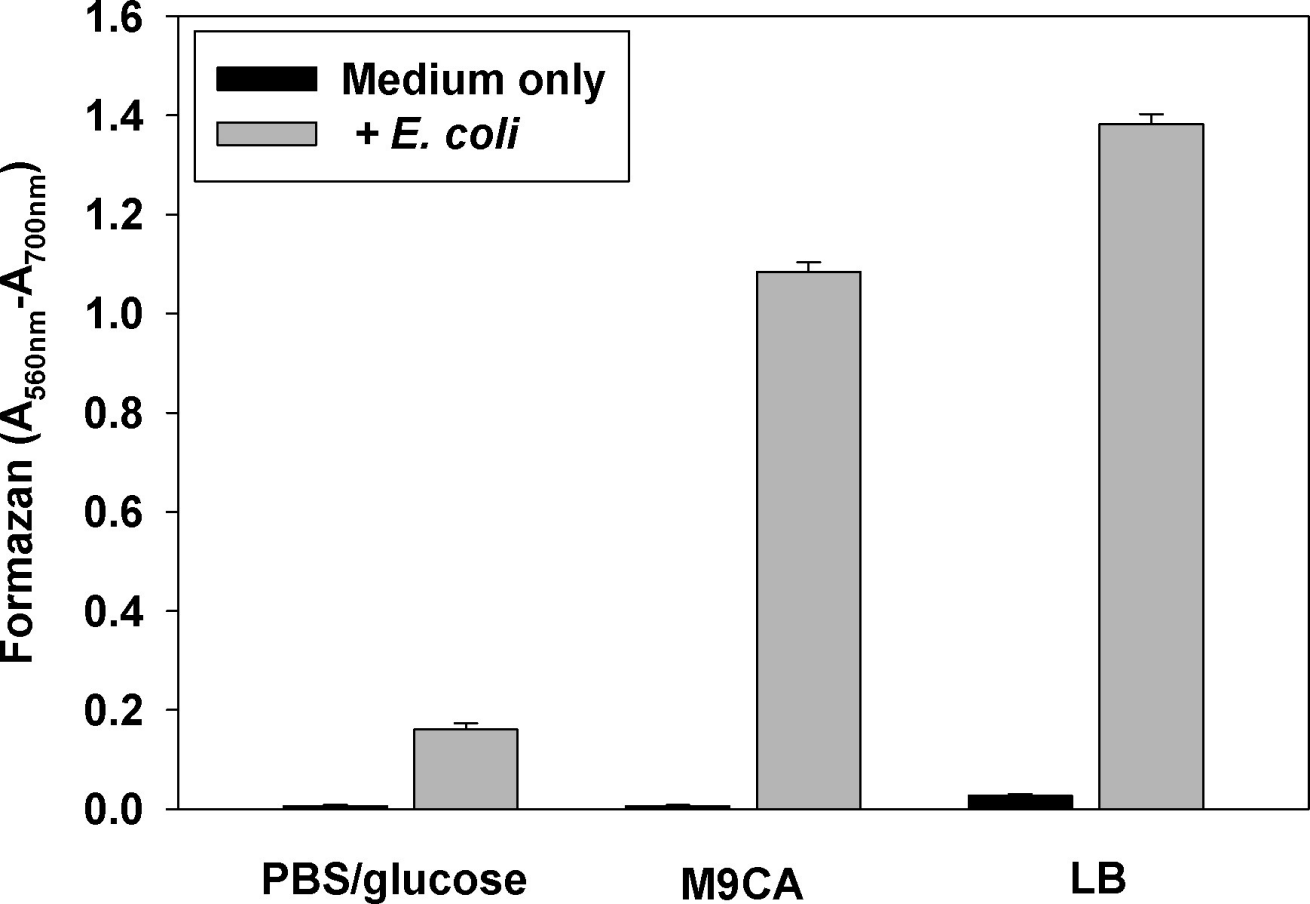
Effect of incubation medium on MTT reduction by *E*. *coli*. *E*. *coli* GC4468 strain was grown to mid-log phase in LB medium, the cells were thoroughly washed and resuspended to OD_600_ = 0.1 in PBS + glucose, M9CA medium, or LB medium. The MTT assay was performed as described in Materials and Methods section and formazan crystals were solubilized with SDS-HCl. Results are expressed as difference between absorbance at 560 nm (formazan peak) and 700 nm (background). Experiments were repeated twice, each sample in triplicates. Mean ± S.D. is presented.

The purpose of the initial experiments was to establish the effect of the composition of incubation medium on reduction of MTT to formazan by bacteria. Results presented in **Fig 1**show that the formation of formazan depended on the medium. The amount of formed formazan was the lowest in PBS + glucose, and the highest in LB medium.

Since formazan crystals are not soluble in water/growth media, a variable that affects the outcome of the assay is the solvent used for solubilization of the crystals [6, 15]. It has been suggested that the composition of the growth medium would affect formazan dissolution and consequently, the reliability of the data. The effect of the solvent on MTT assay outcome was tested using SDS-HCl, SDS in phosphate buffer, DMSO ± SDS, ammonia-DMSO [15], and isopropanol ± SDS. Results demonstrated that:

- Solubilization with SDS-phosphate produced higher formazan absorption peak than solubilization with SDS-HCl (S1A Fig). Such an effect of pH on formazan absorption has been previously reported [16]. It is important to note that solubilization of *E*.*coli*/formazan with SDS-phosphate required mechanical stirring and longer incubation.
- When formazan was solubilized with DMSO, the absorbance at 560 nm (formazan absorption peak [17]) was much lower than when SDS was used (S1A Fig). At the same time, absorbance at 700 nm (far from the formazan peak [17]) reached very high values (S1B Fig). When the wells in the 96-well plates were visually assessed, undissolved formazan crystals and cell aggregates were found (S1C Fig). The crystals were not dissolved even when kept in the solvent for up to 12 hours. Poor solubilization of formazan resulted in negative values of the difference A_560 nm_−A_700 nm_ (S1D Fig) and lack of the typical absorbance peak when absorption spectrum was recorded (S1E Fig). Better dissolution of formazan was observed in isopropanol, but again solubilization was incomplete, and with cultures incubated in LB medium, precipitate could be observed with naked eye (S1C Fig, top row).

Ammonia-DMSO [15] was a better solvent than DMSO alone, giving a good formazan peak and low non-specific absorbance at 700 nm with cultures in PBS-glucose (S1F Fig), but in LB medium the formazan peak appeared shallow and absorbance at 700 nm was higher (S1G Fig).

No difficulties with formazan dissolution have been reported for mammalian cells when organic solvents were used. A possible explanation for poor solubility of formazan produced by bacteria, can be found in the presence of thick polysaccharide layer [18], and differences in formazan crystals. In contrast to mammalian cells which produce needle-shaped crystals [19], formazan crystals formed by *E*. *coli* have different shape and form complexes with the cell [11, 19]. When treated with organic solvents, bacterial cells form clumps which trap formazan and prevent its solubilization. To test this explanation, SDS was added to cell suspensions either before the addition of the organic solvent or mixed with it. As S1B, S1C and S1D Fig show, addition of SDS substantially improved formazan solubility, which is reflected by the decrease in the non-specific absorbance at 700 nm and increase of absorbance at 560 nm.

MTT, at concentrations used in the assay, does not prevent bacteria from proliferating. Because bacterial cultures have different growth rates in different media, it is possible that increased production of formazan in LB and M9CA media compared to PBS/glucose, reflects differences in cell number. In order to eliminate the growth factor, cultures in different media were preincubated with 10 μg/ml chloramphenicol before MTT was added. Judging by OD_600 nm_, chloramphenicol completely blocked cell proliferation. To be sure that chloramphenicol does not interfere with the assay, the MTT test was performed on non-growing, stationary phase cultures with and without addition of chloramphenicol (S2 Fig inset). S2 Fig shows that the difference in formazan formation between non-treated and chloramphenicol-treated cultures was the highest when the assay was performed in LB medium, which reflects the highest rate of cell proliferation in this medium. Addition of chloramphenicol did not affect MTT reduction when cells were resuspended in PBS/glucose (S2 Fig) because no significant increase of cell number was detected in this medium. Even in non-proliferating cultures, MTT reduction to formazan remained the highest in LB medium and the lowest in PBS-glucose.

**Fig 2:**
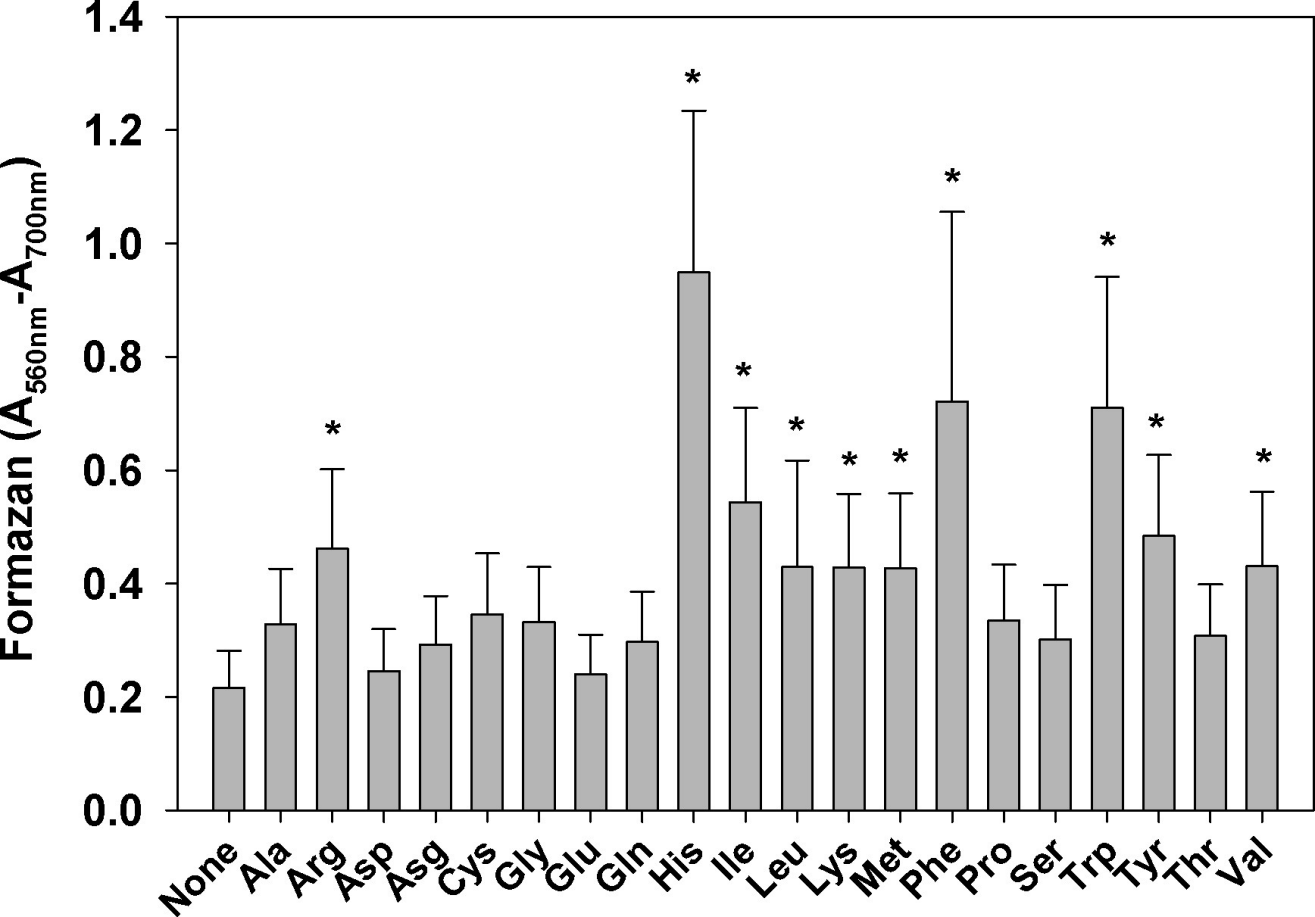
Effect of amino acids on reduction of MTT to formazan by *E*. *coli*. Mid-log LB culture was washed and resuspended in PBS + glucose to OD_600_ = 0.5. One hundred μl aliquots were dispensed in triplicate wells of a 96-well plate, amino acid sterile solutions were added to a final concentration of 5.0 mM, and the MTT test was performed. Controls, containing PBS-glucose and the respective amino acid, but not *E*. *coli*, were run in parallel. The experiment was repeated three times. Results are presented as mean ± SD. *Indicates statistically significant difference compared to controls containing *E*. *coli* in PBS/glucose but not amino acids (p < 0.05).

These results suggest that the presence of amino acids might be the reason for increased formazan production by cells suspended in M9CA or LB media. To test such a possibility, the twenty amino acids commonly present in growth media were individually tested. Results (**Fig 2**) demonstrated that histidine caused the strongest increase of formazan formation by *E*. *coli*. Phenylalanine and tryptophan had a weaker effect, followed by isoleucine, tyrosine, and arginine. Only one amino acid, cysteine, reduced MTT to formazan in the absence of cells. Because no detectable culture growth (increase of OD_600 nm_) was observed during the 30 minutes incubation with MTT, no corrections for cell proliferation were made.

Similar results were obtained when a Gram-positive strain, *S*. *aureus* was tested, except that compared to *E*. *coli*, four amino acids, arginine, leucine, methionine, and phenylalanine, did not show statistically significant increase in MTT reduction to formazan (S3 Fig).

Since histidine was among the most efficient amino acid in promoting MTT reduction by *E*. *coli* and *S*. *aureus*, it was selected to investigate the mechanism of this effect. Addition of histidine to *E*. *coli* or *S*. *aureus* cell suspensions incubated in PBS/glucose, increased MTT reduction by a concentration-dependent manner. MTT reduction reached saturation at 5.0 mM histidine (S4 Fig). In the absence of cells, histidine had negligible effect on MTT reduction (S4A Fig, Inset).

**Fig 3:**
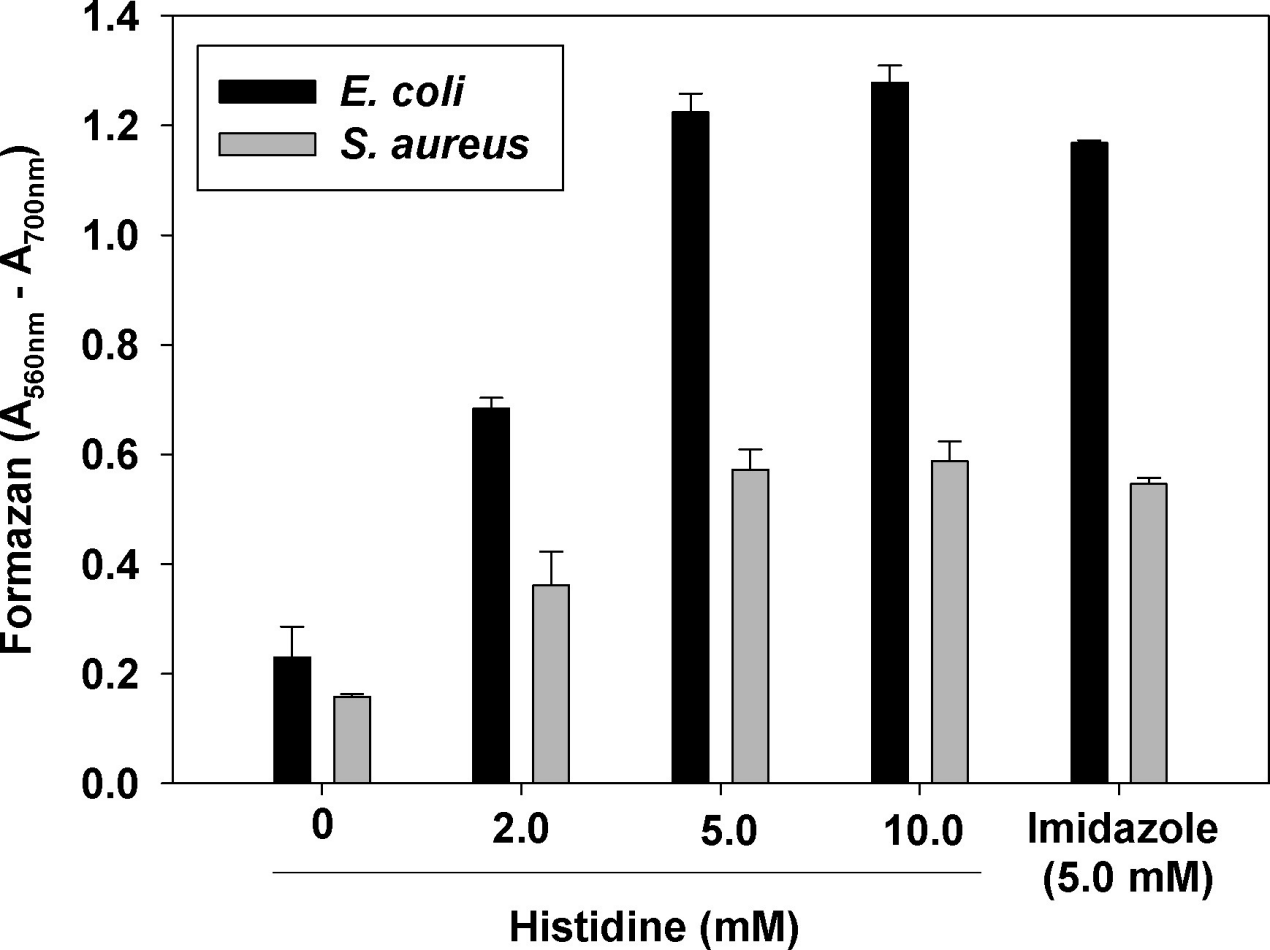
Concentration-dependence of the effect of histidine on MTT reduction. Histidine or imidazole were added to *E*. *coli* and *S*. *aureus* cell suspensions with density of OD_600nm_ = 0.1. In all experiments the medium was PBS + glucose. After 10 min of incubation with histidine or imidazole, the MTT assay was performed. Experiments were repeated three times, each sample in triplicate. Mean ± S.D. is presented.

To test if the effect of histidine was cell density-dependent, the experiment was performed with five-fold lower cell density (OD_600nm_ = 0.1). The same concentration-dependence of the effect of histidine was observed (**Fig 3**). At concentrations above 5.0 mM histidine did not further increase MTT reduction to formazan.

The observation that not all amino acids stimulated bacterial reduction of MTT to formazan, suggests that the effect depends on the amino acid side chain. **Fig 3**shows that imidazole, the side chain of histidine, produced the same effect as histidine.

**Fig 4:**
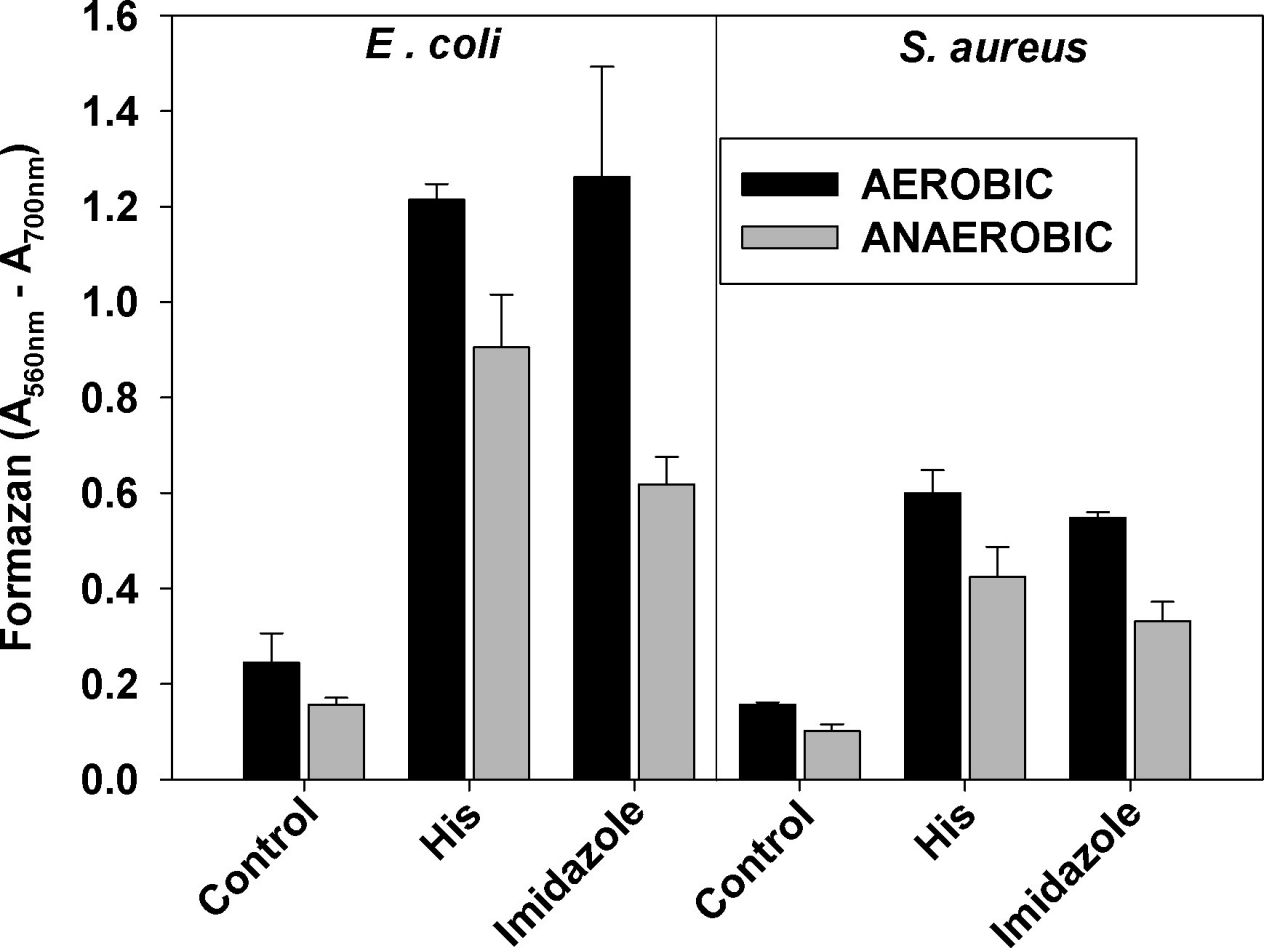
Effect of growth conditions on the increase of formazan production by histidine and imidazole. Mid-log *E*. *coli* and *S*. *aureus* cultures grown aerobically or anaerobically in LB and BHI media, respectively, were washed and diluted in PBS/glucose to OD_600 nm_ = 0.1. Histidine or imidazole were added to a final concentration of 5.0 mM, followed ten minutes later by addition of MTT (0.5 mg/ml). Samples were incubated 60 min at 37°C in a 96-well plates with shaking. At the end of the incubation period, SDS was added, and after 60 min of incubation, absorbance was measured at 560 nm and 700 nm (background). The average of the difference A_560 nm_-A_700 nm_ of two separate experiments, each sample run in triplicate, ± SD is presented.

In contrast to eukaryotic cells, data about mechanism of MTT reduction to formazan by prokaryotic cells is limited, but it is believed that NADH dehydrogenases are involved [6]. It might be expected that growth of bacteria under aerobic or anaerobic conditions would make a difference with respect to the effect of histidine on MTT reduction. **Fig 4**shows that growing *E*. *coli* and *S*. *aureus* cultures under anaerobic conditions and then exposing them aerobically to histidine or imidazole, produced results qualitatively similar to results obtained when cultures were grown aerobically.

**Fig 5:**
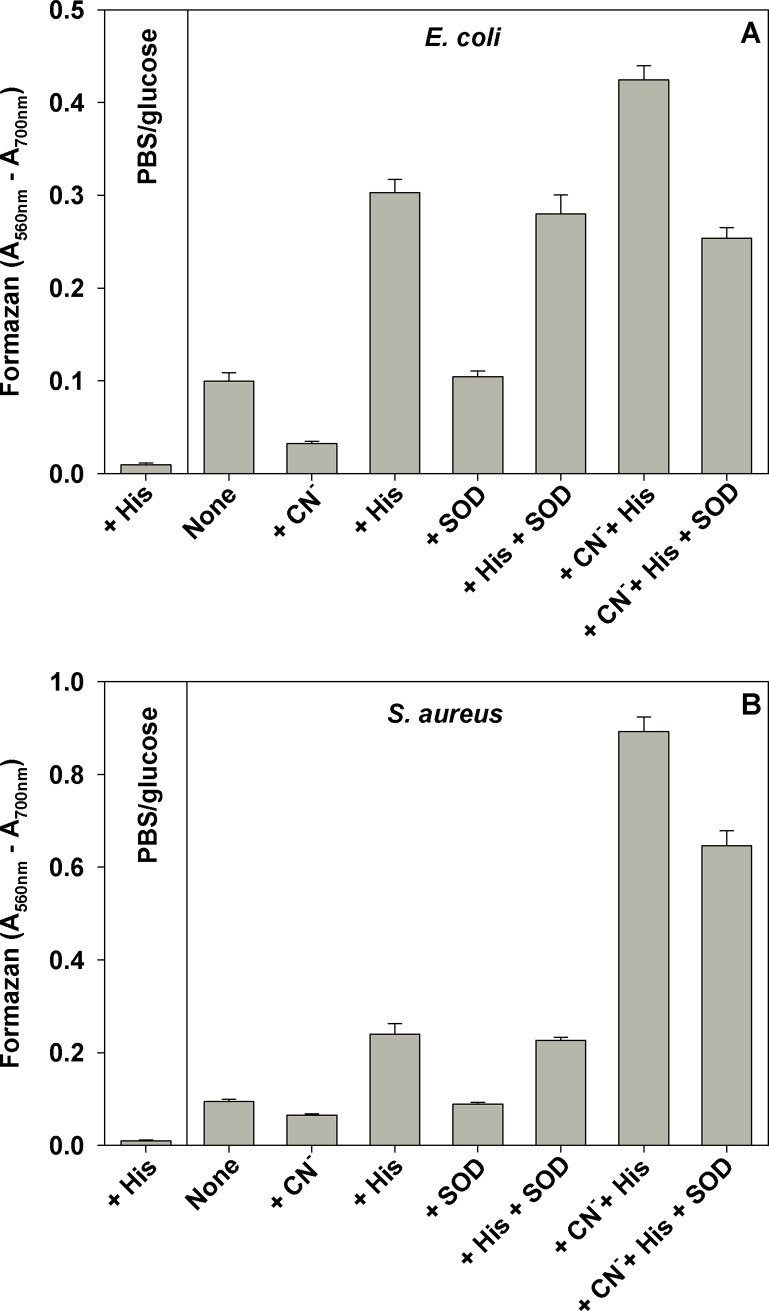
Effect of CN^-^ and SOD on histidine-induced formazan formation. *E*. *coli* and *S*. *aureus* cultures were grown aerobically to mid-log phase in LB/BHI media respectively. Cells were washed and resuspended in PBS/glucose. Histidine and KCN were added to 5.0 mM, SOD was 400 U/ml, and MTT was 0.5 mg/ml. Samples in triplicate were incubated 30 min at 37 ^o^C on a shaker at 200 rpm/min. At the completion of the incubation, equal volume (100 μl) of 10% SDS-HCl was added in each well and absorbance was measured at 560 and 700 nm. The average of the difference A_560 nm_-A_700 nm_ of two separate experiments, each sample run in triplicate, ± SD is presented.

Among the possible reasons for increased formazan formation by histidine could be increased production of superoxide anion radical [12]. Cyanide increases superoxide production by blocking the electron flow in the respiratory chain, thus leading to accumulation of reduced electron carriers and other electron transport chain components [20]. Addition of KCN suppressed MTT reduction by *E*. *coli* in the absence of histidine, but had the opposite effect if histidine was present (**Fig 5A**). Addition of superoxide dismutase (SOD) to *E*. *coli* cell suspensions had no effect on MTT reduction irrespective of the absence or presence of histidine. Addition of SOD to cell suspensions where KCN and histidine were simultaneously present, suppressed MTT reduction by about 40% (**Fig 5A**). Similar result was obtained with *S*. *aureus*, except that the effect of CN^-^ on histidine-stimulated MTT reduction was much stronger (**Fig 5B**).

**Fig 6:**
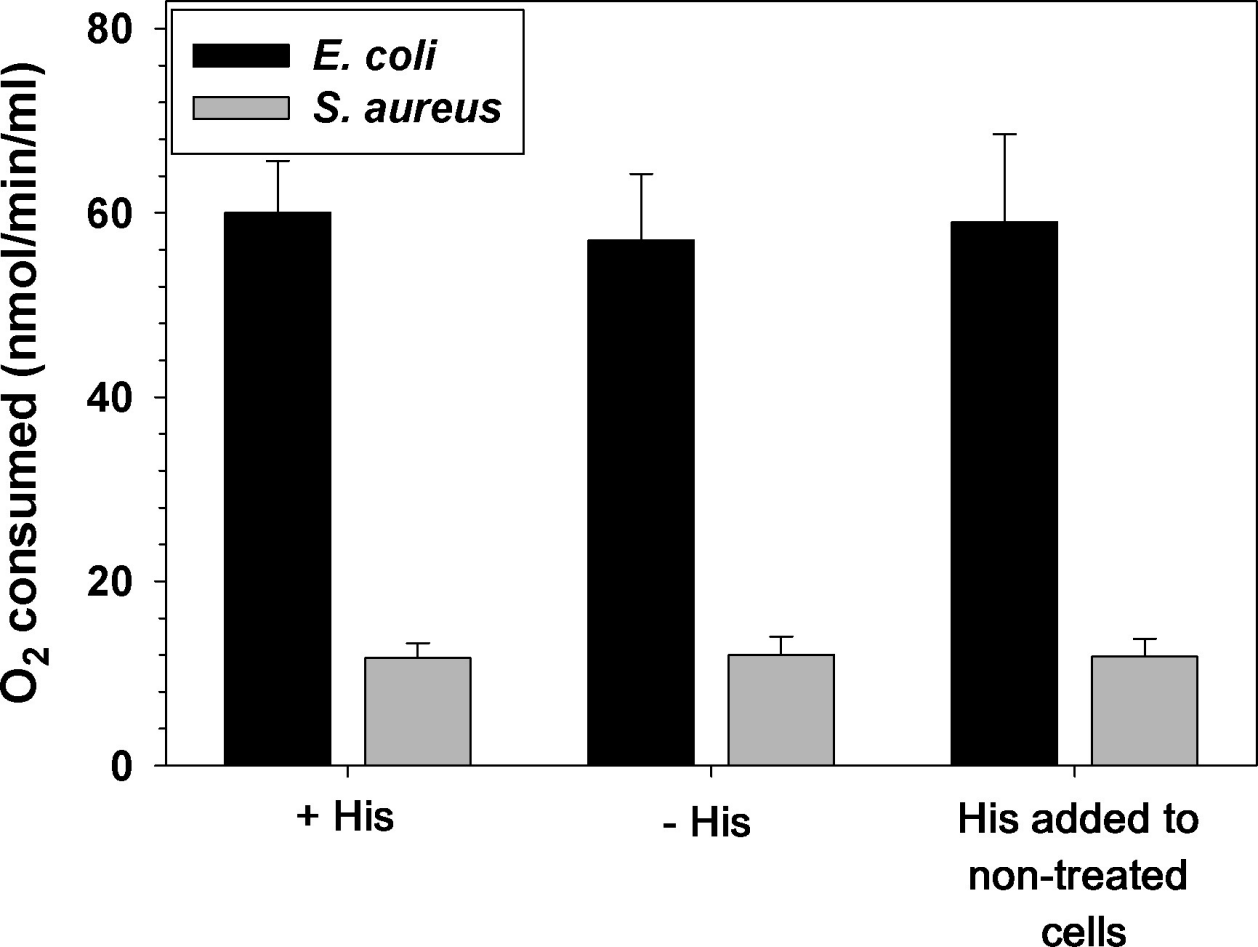
Effect of histidine on respiration. *E*. *coli* and *S*. *aureus* were grown to mid-log phase with 5.0 mM histidine (His) or without histidine. The cells were washed and resuspended to OD = 0.5 in PBS/glucose. Half ml of cell suspension was added to 2.5 ml of PBS glucose in a thermostatic chamber fitted with a Clark electrode. In separate runs, histidine was added to non-treated cell suspensions to a final concentration of 5.0 mM (the third set of bars). The experiment was repeated twice in triplicates. Mean ± SD is presented.

The enhancement of the effect of histidine by CN^-^ suggests that histidine either diverts electrons from the electron transport chain towards MTT or affects cellular respiration. To test the second possibility, oxygen consumption by *E*. *coli* and *S*. *aureus* cultures incubated with and without histidine was measured. Results demonstrated that incubation of bacterial cultures with histidine did not affect respiration. Adding histidine to bacterial suspensions in the Clark electrode camber also did not have any effect on oxygen consumption (**Fig 6**).

**Fig 7:**
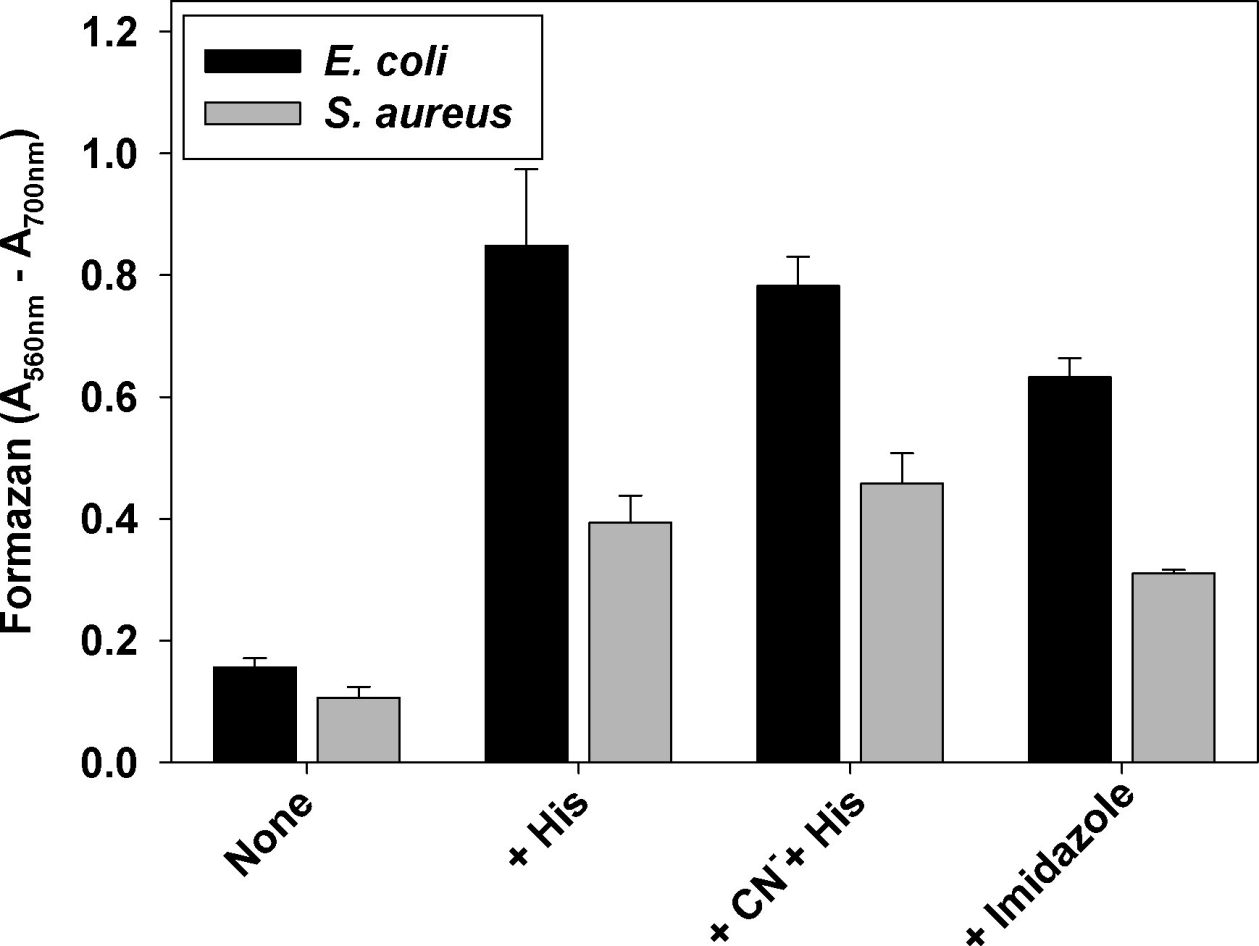
Effect of histidine on MTT reduction under anaerobic conditions. *E*. *coli* and *S*. *aureus* cultures were grown aerobically to mid-log phase in LB/BHI media respectively. Cells were washed and resuspended in PBS/glucose. The cell suspension was aliquoted in 96-well plates and transferred to anaerobic Coy chamber. The cultures were equilibrated, and degased solutions of histidine (5.0 mM), KCN (5.0 mM), or imidazole (5.0 mM), were added, followed by addition of MTT solution to all wells. After 60 min of incubation at 37 ^o^C, SDS solution was added, plates were taken out, and absorbance at 560 and 700 nm was measured. The average of the difference A_560 nm_-A_700 nm_ of two separate experiments, each sample run in triplicate, ± SD is presented.

To test if the effect of histidine is oxygen-dependent, aerobically grown cultures were transferred to anaerobic Coy chamber and MTT reduction was carried out under anaerobic conditions. Results (**Fig 7**) demonstrated that the lack of oxygen did not abolish the effect of histidine or imidazole.

**Fig 8:**
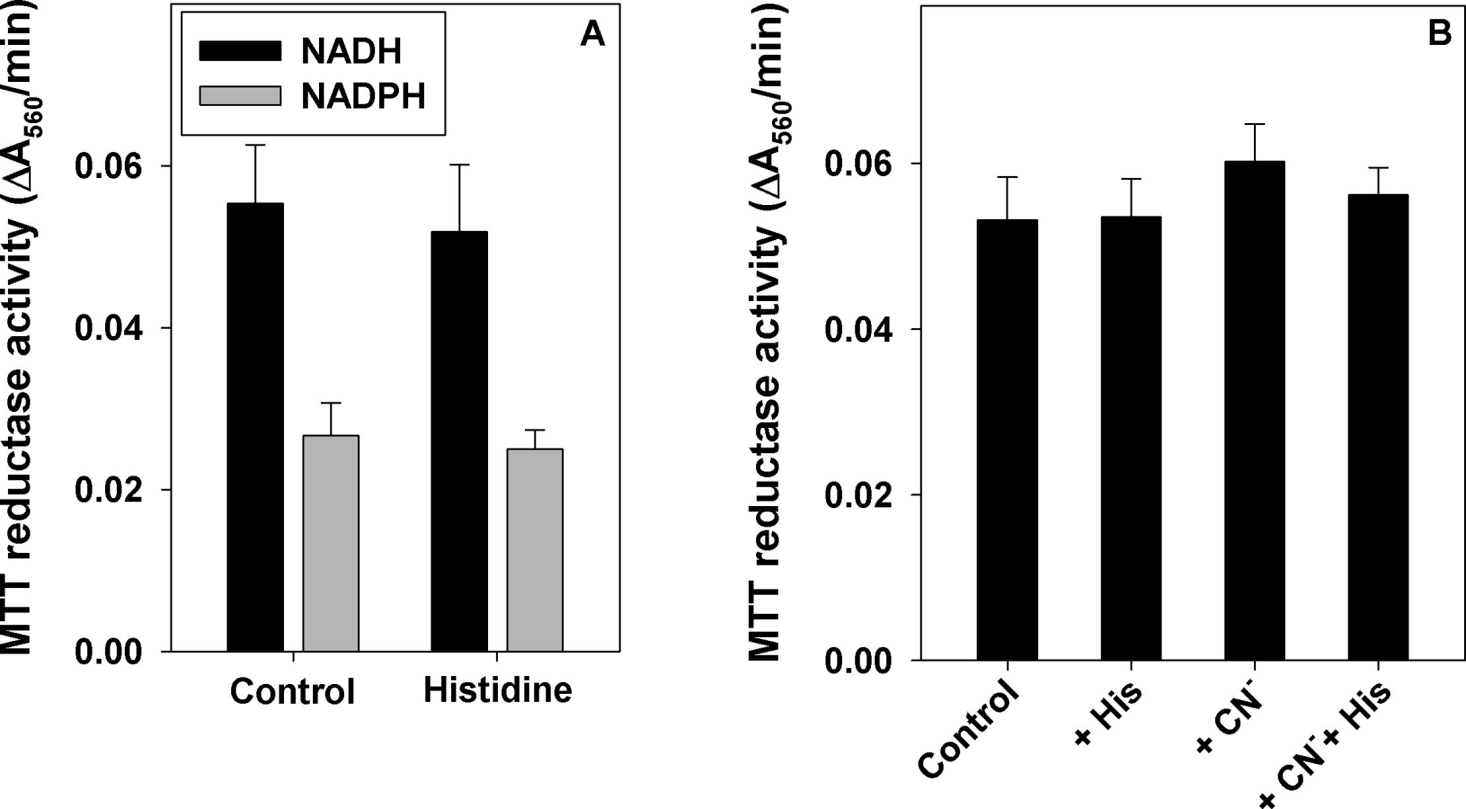
Reduction of MTT to formazan by cell-free extracts. Mid-log *E*. *coli* (OD_600_ = 0.5) was incubated with or without 5.0 mM histidine for 60 min at 37 ^o^C in PBS/glucose. The cells were washed and disrupted by French press. MTT reductase activity was determined spectrophotometrically by measuring formation of formazan at 560 nm. Reaction mixture contained 0.5 mg MTT, and 3.5 mM NADH or NADPH in one ml of 50 mM phosphate buffer, pH 7.4. Reaction was started by adding cell-free extract. The experiment was repeated three times. Mean ± SD is presented.

Since MTT reduction is catalyzed by dehydrogenases, another possible explanation of the effect of histidine would be induction of dehydrogenase(s) participating in MTT reduction. To test such a possibility, after incubation with histidine, *E*. *coli* cultures were washed and disrupted, and MTT reduction by cell-free extracts was determined with NADH and NADPH as electron donors (**Fig 8**). No difference in MTT reduction by cell-free extracts obtained from cells incubated with and without histidine were observed (**Fig 8A**). Preincubation with chloramphenicol also did not abolish the effect of histidine (not shown). These results rule out induction by histidine of enzyme(s) reducing MTT at the expense of NADH or NADPH. Addition of histidine or KCN to cell-free extracts also had no effect on MTT reduction (**Fig 8B**).

Based on results obtained so far, it appears that histidine does not induce genes coding for enzymes reducing MTT and does not affect respiration. No evidence for metabolic alterations caused by histidine was found.

**Fig 9:**
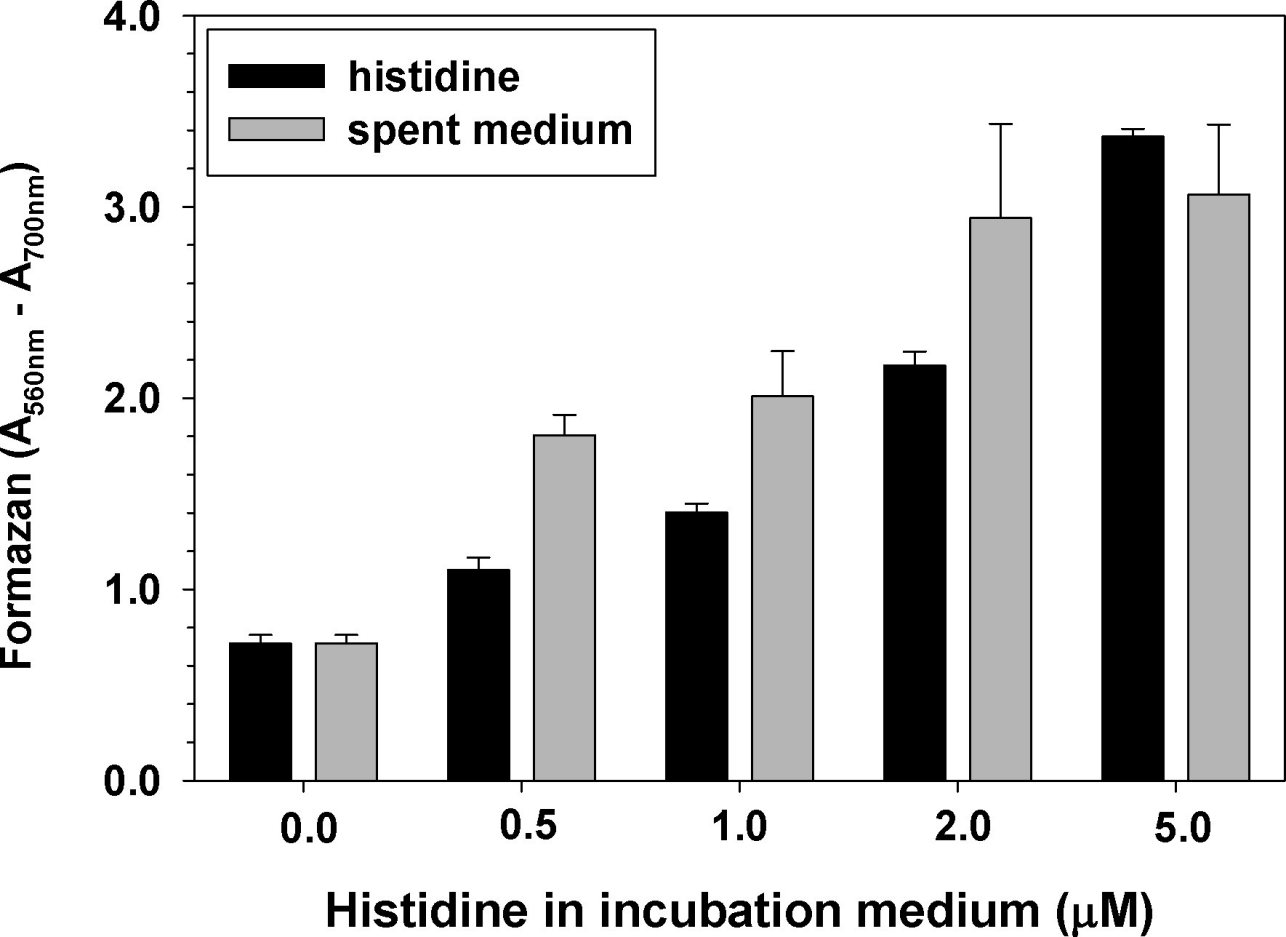
Effect of spent histidine medium on bacterial reduction of MTT to formazan. Mid-log *E*. *coli* (OD_600_ = 0.5) was thoroughly washed with PBS and resuspended in PBS containing 0.2% glucose and histidine at the concentration range 0.5–5.0 mM. After 12 hours of incubation at 37 ^o^C on a shaking water bath, cultures were centrifuged and 10 μl aliquots of the cell-free spent medium were added to 90 μl fresh mid-log *E*. *coli* suspension in PBS/glucose in a 96-well plate. To a separate set of wells containing *E*. *coli* in PBS/glucose, freshly prepared histidine solution was added to final concentrations 0.5–5.0 mM. Since the spent medium was diluted tenfold, the corresponding histidine concentration for the spent medium is 10x lower. The experiment was repeated three times, each sample in triplicate. Mean ± SD is presented.

Incubation of metabolically active *E*. *coli* in a medium containing histidine led to substantial change in the UV spectrum of histidine, reflecting the appearance of metabolic product(s) (S5 Fig). No such changes were observed when the culture was kept for the same time at 4°C. It can be speculated that metabolic product(s) derived from histidine, facilitate delivery of electrons to MTT, thus stimulating formazan formation. In an attempt to test this hypothesis, *E*. *coli* was incubated for 12 hours in a medium containing PBS, glucose, and varying concentrations of histidine. After separating the medium from the cells, the effect of the spent cell-free medium on formazan formation by fresh *E*. *coli* cultures was tested. **Fig 9**shows that for each histidine concentration tested, the heights of the bars reflecting formation of formazan in the presence of fresh histidine and spent histidine medium, are comparable. Since only 10 μl spent medium was added per 100 μl well, it appears that spent medium is about tenfold more active in stimulating formazan formation by *E*. *coli* than is fresh histidine. In the absence of cells, spent medium had negligible effect on MTT reduction (S6 Fig).

Metabolism of histidine by bacteria has been studied in detail and it is known that the two pathways involved, produce either one mole of ammonia, glutamate, and formate (Pathway 1) or two moles of ammonia, one mole of glutamate, and one mole of formate (Pathway 2) per mole of histidine [21]. **Fig 2**shows, that one of these metabolites, glutamate, had no effect on MTT reduction. When the other three, ammonia, formate, and formamide were tested, it was found that only formate increased MTT reduction by *E*. *coli*, but its effect was smaller than that of equimolar histidine (S7 Fig).

In conclusion, results of this study show that bacterial reduction of MTT to formazan is strongly influenced by the composition of the medium. Some of the tested twenty amino acids more than doubled formazan production, with histidine having the highest activity. The effect of histidine seems to be mediated by metabolic product(s) released by bacteria. Even though the exact nature of the product(s) has not been determined, it becomes clear that data obtained with the MTT test should be interpreted with caution, particularly when different growth media are used or treatments affect metabolic pathways. Another variable that influences MTT assay outcome is the rate of cell division during incubation with MTT.

The best way to avoid influence of such variables on formazan production would be to perform the assay in a defined medium like PBS glucose, which eliminates the effects of amino acid metabolites and limits cell division during incubation with MTT. This, however, is not always possible. Furthermore, it adds time consuming steps of washing and resuspending the cells in a new medium, which eliminates some of the main advantages of the MTT assay. Nevertheless, evaluation of the reliability of the MTT assay under specific conditions should be performed to avoid erroneous results. Another critical element to be considered is the choice of a proper solvent for dissolution of formazan crystals. Incomplete solubilization or distortion of formazan absorption spectrum would inevitably alter assay outcome. Even well established and recommended solvents may produce unsatisfactory results when particular microorganisms, media, or additives are tested.

## Supporting information

S1 Fig Effect of solvents on formazan recovery. *E*. *coli* GC4468 strain was grown to mid-log phase in LB medium, the cells were thoroughly washed and resuspended to OD_600_ = 0.1 in PBS + glucose, M9CA, or LB medium. The MTT assay was performed and formazan crystals were dissolved in SDS-HCl, SDS-phosphate (pH 7.4), DMSO, ammonia-DMSO, SDS-DMSO, isopropanol or SDS-isopropanol. **Panel A**: absorbance at formazan peak; **Panel B**: non-specific absorbance away from the peak; **Panel C**: wells of a 96-well plate showing undissolved formazan; **Panel D**: Formazan amount presented as a difference between the absorbance at the peak and background absorbance resulting from turbidity and undissolved formazan; **Panel E**: typical absorption spectrum obtained when MTT assay was perfumed for *E*. *coli* in LB medium and formazan was solubilized with DMSO; **Panel F**: absorption spectrum of formazan formed by *E*. *coli* in PBS-glucose, dissolved with ammonia-DMSO; **Panel G**: absorption spectrum of formazan formed by *E*. *coli* in LB medium, dissolved with ammonia-DMSO.
Experiments were repeated twice, each sample in triplicates. Bars show mean ± S.D.
(PDF)

S2 Fig MTT reduction to formazan by growing and non-growing *E*. *coli* cultures. *E*. *coli* GC4468 strain was grown to mid-log phase in LB medium, the cells were thoroughly washed and resuspended to OD_600_ = 0.1 in PBS + glucose, M9CA, or LB media. To half of the culture flasks chloramphenicol was added to a final concentration of 10 μg/ml. After 15 minutes of incubation at 37 ^o^C at 200 RPM, OD_600 nm_ was checked again and where necessary, was adjusted to 0.1. Hundred μl aliquots were distributed in 96-well plates and the MTT assay was performed. Separate sets of identical wells were kept without MTT for monitoring changes in OD_600 nm_.
**Inset:** Stationary phase cultures treated with chloramphenicol as above.
The experiment was repeated two times in triplicates. Results are presented as mean ± SD.
(PDF)

S3 Fig Effect of amino acids on reduction of MTT to formazan by *S*. *aureus*. Mid-log culture was washed and resuspended in PBS + glucose to OD_600_ = 0.5. One hundred μl aliquots were dispensed in triplicate well of a 96-well plate, respective amino acid sterile solutions were added to a final concentration of 5.0 mM, and the MTT test was performed. Controls containing PBS-glucose, and the respective amino acid, but not *S*. *aureus*, were run in parallel. The experiment was repeated three times. Results are presented as mean ± SD. *Indicates statistically significant difference compared to controls containing *S*. *aureus* but not amino acids (p < 0.05).
(PDF)

S4 Fig **Concentration-dependent effect of histidine on formazan formation by *E*. *coli* (Panel A) and *S*. *aureus* (Panel B).** Histidine was added to mid-log cell suspensions (OD_600_ = 0.5) or to PBS + glucose (Inset. Note that the Y axis is expanded 100 fold) and the MTT test was performed. Experiment was repeated three times, each with three replicates. Mean ± SD is presented.
(PDF)

S5 Fig Changes in the absorption spectrum of histidine as a result of incubation with metabolically active *E*. *coli*. Mid-log *E*. *coli* cultures (OD_600_ = 0.5–0.7) grown in LB medium were thoroughly washed and the cells were resuspended to the initial volume in PBS/glucose medium containing 5.0 mM histidine. After 12 hours of incubation on a shaking water bath at 37 ^o^C and 200 rpm, cultures were centrifuged, cell-free medium was diluted 30x with deionized water and absorption spectra were recorded. Controls containing PBL/glucose and 5.0 mM histidine were incubated under the same conditions. Line 1, control; line 2, incubated with *E*. *coli*.
The experiment was repeated three times with similar outcome. The graph shows original record of a single experiment. The arrow points to a new peak at 273.5 nm.
(PDF)

S6 Fig Effect of spent histidine medium on formazan formation in the absence of cells. Spent medium was prepared by incubating mid log *E*. *coli* cultures in PBS/glucose, containing the indicated histidine concentrations. After 12 hours of incubation at 37 ^o^C on a shaking water bath, cultures were centrifuged and cell-free spent medium was aliquoted in a 96-well plate (100 μl/well). Medium not incubated with cells was used for comparison. The experiment was repeated three times, each sample in triplicate. Mean ± SD is presented.
(PDF)

S7 Fig Effect of histidine metabolic products on MTT reduction to formazan by *E*. *coli*. The cells of mid-log LB culture were thoroughly washed and resuspended to OD_600_ = 0.1 in PBS + glucose. Hundred μl aliquots were distributed to triplicate wells in 96-well plates, formamide, ammonium formate, ammonia, and histidine were added to a final concentration of 5.0 mM, and after 30 minutes of incubation, the MTT test was performed. The experiment was repeated two times, each sample run in triplicate. Mean ± SD is presented.
(PDF)
