# Supplementary material for: Effect of growth media on the MTT colorimetric assay in bacteria
Source: PLoS One. 2019 Aug 27;14(8):e0219713. doi: 10.1371/journal.pone.0219713 (PMC6711527; doi:10.1371/journal.pone.0219713)
Supplement: S1 Fig — E. coli GC4468 strain was grown to mid-log phase in LB medium, the cells were thoroughly washed and resuspended to OD600 = 0.1 in PBS + glucose, M9CA, or LB medium. The MTT assay was performed and formazan crystals were dissolved in SDS-HCl, SDS-phosphate (pH 7.4), DMSO, ammonia-DMSO, SDS-DMSO, isopropanol or SDS-isopropanol. Panel A: absorbance at formazan peak; Panel B: non-specific absorbance away from the peak; Panel C: wells of a 96-well plate showing undissolved formazan; Panel D: Formazan amount presented as a difference between the absorbance at the peak and background absorbance resulting from turbidity and undissolved formazan; Panel E: typical absorption spectrum obtained when MTT assay was perfumed for E. coli in LB medium and formazan was solubilized with DMSO; Panel F: absorption spectrum of formazan formed by E. coli in PBS-glucose, dissolved with ammonia-DMSO; Panel G: absorption spectrum of formazan formed by E. coli in LB medium, dissolved with ammonia-DMSO. Experiments were repeated twice, each sample in triplicates. Bars show mean ± S.D. (PDF) [file pone.0219713.s001.pdf]

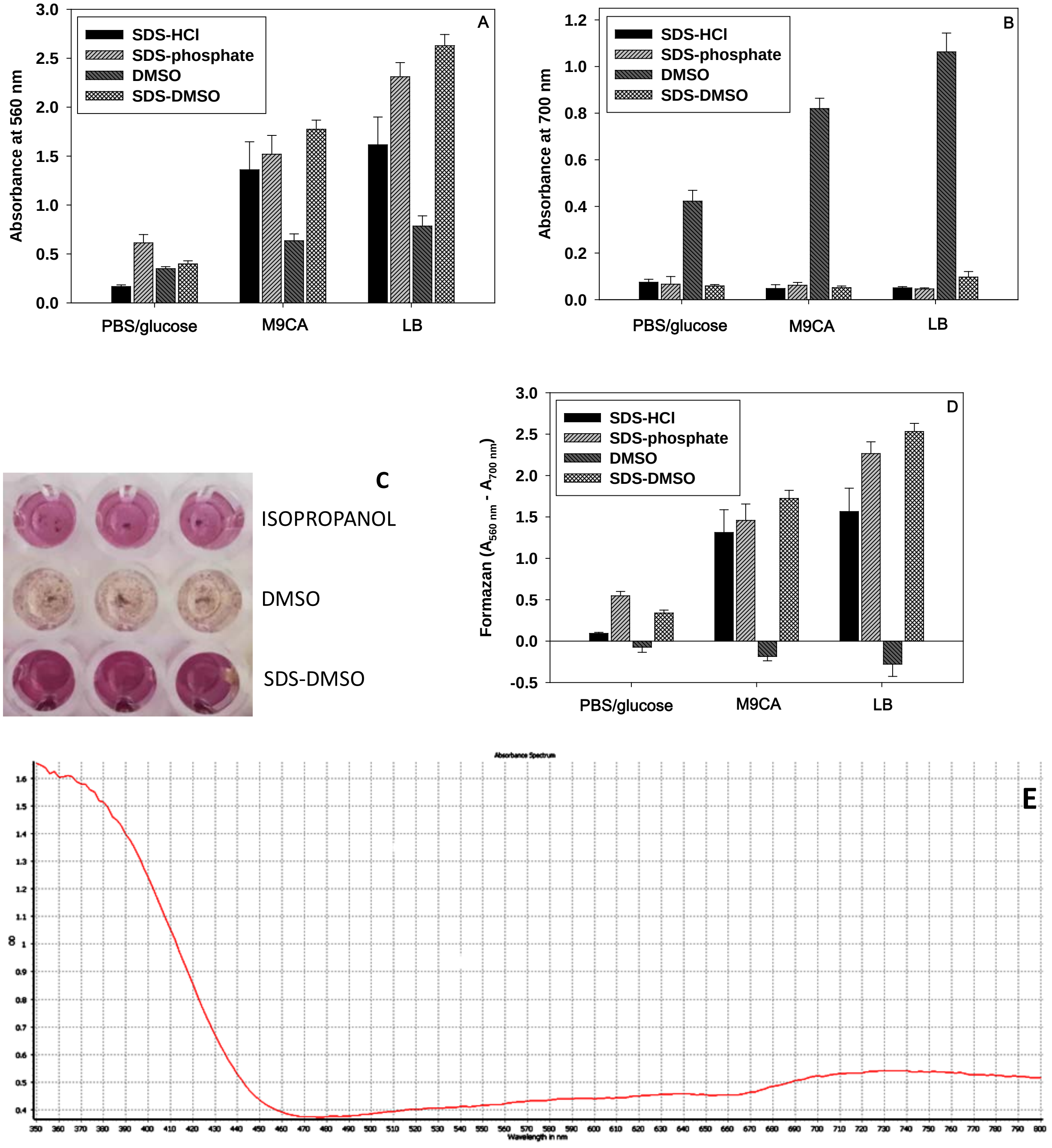
A
Absorbance at 560 nm
3.0
2.5
2.0
1.5
1.0
0.5
0.0
SDS-HCl
SDS-phosphate
DMSO
SDS-DMSO
PBS/glucose
M9CA
LB
B
Absorbance at 700 nm
1.2
1.0
0.8
0.6
0.4
0.2
0.0
SDS-HCl
SDS-phosphate
DMSO
SDS-DMSO
PBS/glucose
M9CA
LB
C
ISOPROPANOL
DMSO
SDS-DMSO
D
Formazan ($A_{560\ nm}$ - $A_{700\ nm}$)
3.0
2.5
2.0
1.5
1.0
0.5
0.0
-0.5
SDS-HCl
SDS-phosphate
DMSO
SDS-DMSO
PBS/glucose
M9CA
LB
E
Absorbance Spectrum
Wavelength in nm

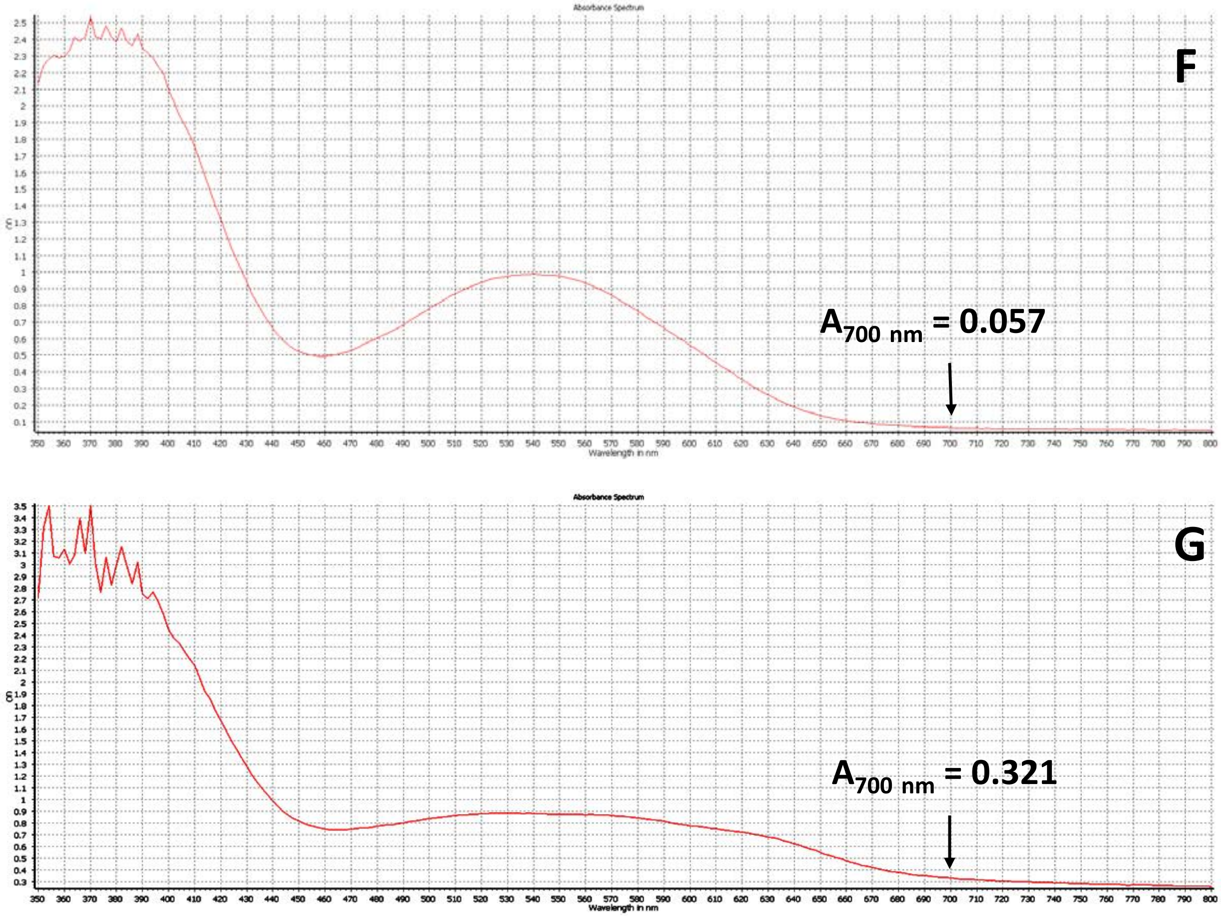

S1 Fig. Effect of solvents on formazan recovery. *E. coli* GC4468 strain was grown to mid-log phase in LB medium, the cells were thoroughly washed and resuspended to OD_{600} = 0.1 in PBS + glucose, M9CA, or LB medium. The MTT assay was performed and formazan crystals were dissolved in SDS-HCl, SDS-phosphate (pH 7.4), DMSO, ammonia-DMSO, SDS-DMSO, isopropanol or SDS-isopropanol. **Panel A**: Absorbance at formazan peak; **Panel B**: Non-specific absorbance away from the peak; **Panel C**: Wells of a 96-well plate showing undissolved formazan; **Panel D**: Formazan amount presented as a difference between the absorbance at the peak and background absorbance resulting from turbidity and undissolved formazan; **Panel E**: Absorption spectrum obtained when MTT assay was perfumed for *E. coli* in LB medium and formazan was solubilized with DMSO; **Panel F**: Absorption spectrum of formazan formed by *E. coli* in PBS-glucose, dissolved with ammonia-DMSO; **Panel G**: Absorption spectrum of formazan formed by *E. coli* in LB medium, dissolved with ammonia-DMSO.
Experiments were repeated twice, each sample in triplicates. Bars show mean ± S.D.
