# Supplementary material for: Effect of growth media on the MTT colorimetric assay in bacteria
Source: PLoS One. 2019 Aug 27;14(8):e0219713. doi: 10.1371/journal.pone.0219713 (PMC6711527; doi:10.1371/journal.pone.0219713)
Supplement: S2 Fig — E. coli GC4468 strain was grown to mid-log phase in LB medium, the cells were thoroughly washed and resuspended to OD600 = 0.1 in PBS + glucose, M9CA, or LB media. To half of the culture flasks chloramphenicol was added to a final concentration of 10 μg/ml. After 15 minutes of incubation at 37 oC at 200 RPM, OD600 nm was checked again and where necessary, was adjusted to 0.1. Hundred μl aliquots were distributed in 96-well plates and the MTT assay was performed. Separate sets of identical wells were kept without MTT for monitoring changes in OD600 nm. Inset: Stationary phase cultures treated with chloramphenicol as above. The experiment was repeated two times in triplicates. Results are presented as mean ± SD. (PDF) [file pone.0219713.s002.pdf]

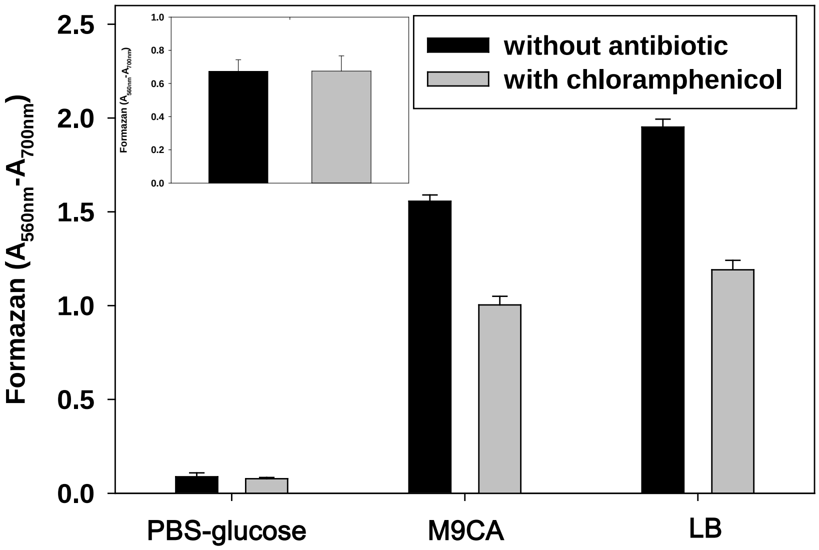

S2 Fig. MTT reduction to formazan by growing and non-growing *E. coli* cultures. *E. coli* GC4468 strain was grown to mid-log phase in LB medium, the cells were thoroughly washed and resuspended to OD_{600} = 0.1 in PBS + glucose, M9CA, or LB media. To half of the culture flasks chloramphenicol was added to a final concentration of 10 µg/ml. After 15 minutes of incubation at 37 ^{o}C at 200 RPM, $OD_{600\ nm}$ was checked again and where necessary, was adjusted to 0.1. Hundred µl aliquots were distributed in 96-well plates and the MTT assay was performed. Separate sets of identical wells were kept without MTT for monitoring changes in $OD_{600\ nm}$.
Inset: Stationary phase cultures treated with chloramphenicol as above.
The experiment was repeated two times in triplicates. Results are presented as mean ± SD.
