# Supplementary material for: Effect of growth media on the MTT colorimetric assay in bacteria
Source: PLoS One. 2019 Aug 27;14(8):e0219713. doi: 10.1371/journal.pone.0219713 (PMC6711527; doi:10.1371/journal.pone.0219713)
Supplement: S3 Fig — Mid-log culture was washed and resuspended in PBS + glucose to OD600 = 0.5. One hundred μl aliquots were dispensed in triplicate well of a 96-well plate, respective amino acid sterile solutions were added to a final concentration of 5.0 mM, and the MTT test was performed. Controls containing PBS-glucose, and the respective amino acid, but not S. aureus, were run in parallel. The experiment was repeated three times. Results are presented as mean ± SD. *Indicates statistically significant difference compared to controls containing S. aureus but not amino acids (p < 0.05). (PDF) [file pone.0219713.s003.pdf]

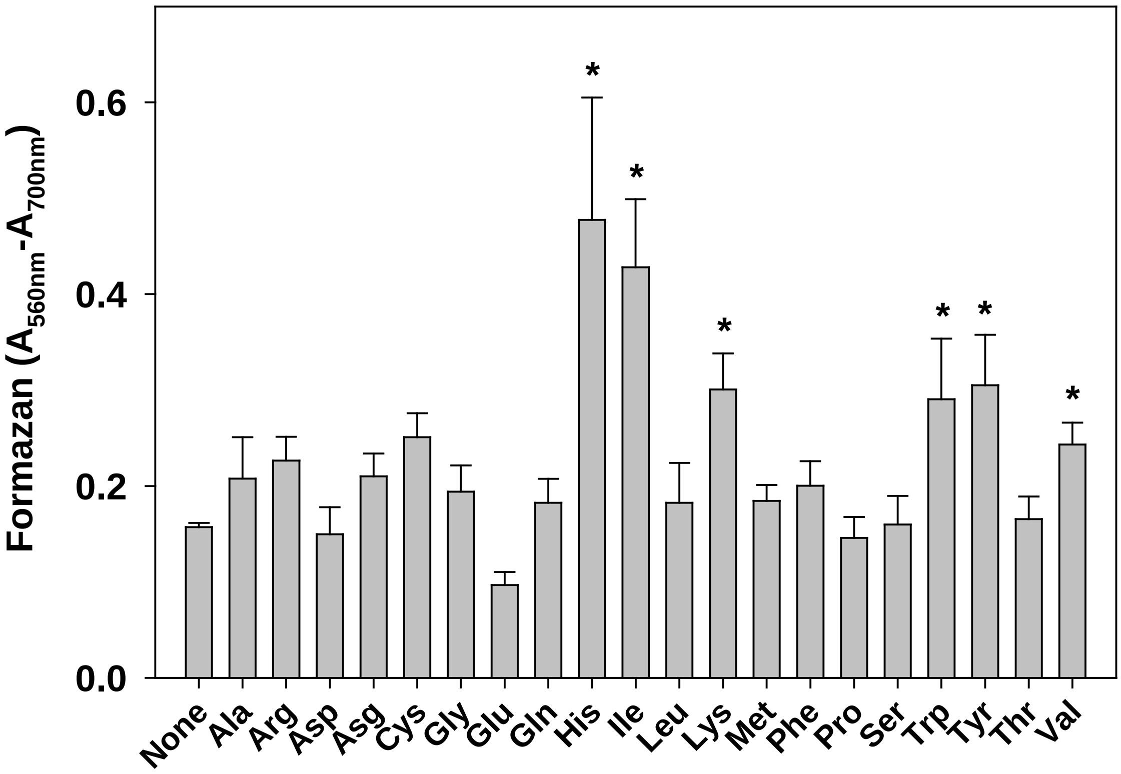

S3 Fig. Effect of amino acids on reduction of MTT to formazan by *S. aureus*. Mid-log culture was washed and resuspended in PBS + glucose to $OD_{600} = 0.5$. One hundred µl aliquots were dispensed in triplicate well of a 96-well plate, respective amino acid sterile solutions were added to a final concentration of 5.0 mM, and the MTT test was performed. Controls containing PBS-glucose, and the respective amino acid, but not *S. aureus*, were run in parallel. The experiment was repeated three times. Results are presented as mean ± SD. *Indicates statistically significant difference compared to controls containing *S. aureus* in PBS/glucose but not amino acids ($p < 0.05$)
