# Supplementary material for: Effect of growth media on the MTT colorimetric assay in bacteria
Source: PLoS One. 2019 Aug 27;14(8):e0219713. doi: 10.1371/journal.pone.0219713 (PMC6711527; doi:10.1371/journal.pone.0219713)
Supplement: S4 Fig — Concentration-dependent effect of histidine on formazan formation by E. coli (Panel A) and S. aureus (Panel B). Histidine was added to mid-log cell suspensions (OD600 = 0.5) or to PBS + glucose (Inset. Note that the Y axis is expanded 100 fold) and the MTT test was performed. Experiment was repeated three times, each with three replicates. Mean ± SD is presented. (PDF) [file pone.0219713.s004.pdf]

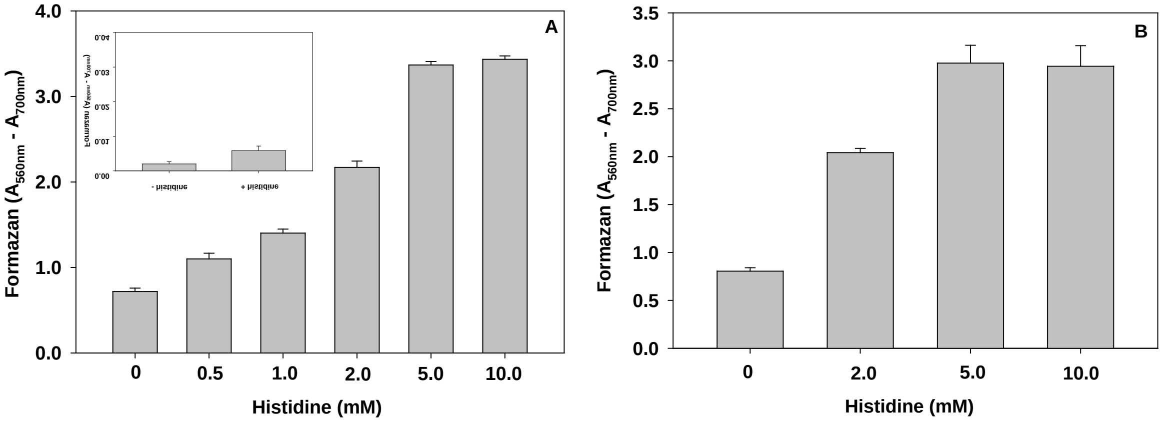


**S4 Fig. Concentration-dependent effect of histidine on formazan formation by *E. coli* (Panel A) and *S. aureus* (Panel B).** Histidine was added to mid-log cell suspensions ($OD_{600}$ = 0.5) or to PBS + glucose (Inset. Note that the Y axis is expanded 100 fold) and the MTT test was performed. The MTT assay was performed and formazan crystals were dissolved in SDS-HCl. Experiment was repeated three times, each with three replicates. Mean ± SD is presented.
