# Supplementary material for: Effect of growth media on the MTT colorimetric assay in bacteria
Source: PLoS One. 2019 Aug 27;14(8):e0219713. doi: 10.1371/journal.pone.0219713 (PMC6711527; doi:10.1371/journal.pone.0219713)
Supplement: S5 Fig — Mid-log E. coli cultures (OD600 = 0.5–0.7) grown in LB medium were thoroughly washed and the cells were resuspended to the initial volume in PBS/glucose medium containing 5.0 mM histidine. After 12 hours of incubation on a shaking water bath at 37 oC and 200 rpm, cultures were centrifuged, cell-free medium was diluted 30x with deionized water and absorption spectra were recorded. Controls containing PBL/glucose and 5.0 mM histidine were incubated under the same conditions. Line 1, control; line 2, incubated with E. coli. The experiment was repeated three times with similar outcome. The graph shows original record of a single experiment. The arrow points to a new peak at 273.5 nm. (PDF) [file pone.0219713.s005.pdf]

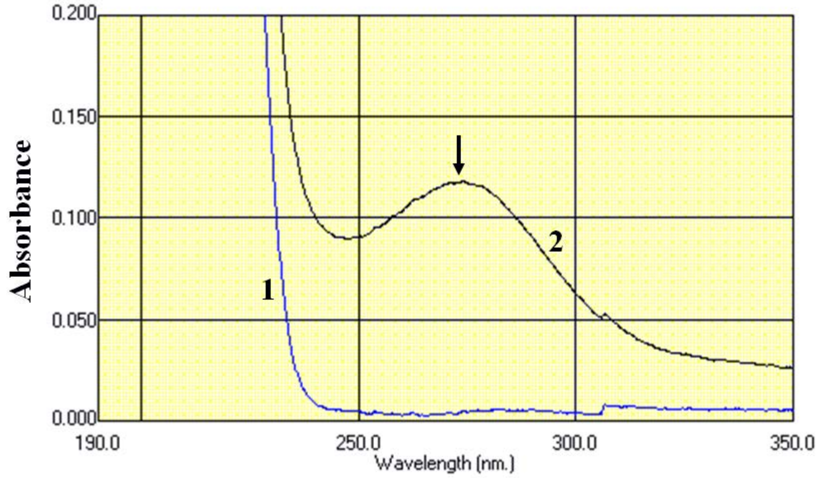

S5 Fig. Changes in the absorption spectrum of histidine as a result of incubation with metabolically active *E. coli*. Mid-log *E. coli* cultures (OD_{600} = 0.5 – 0.7) grown in LB medium were thoroughly washed and the cells were resuspended to the initial volume in PBS/glucose medium containing 5.0 mM histidine. After 12 hours of incubation on a shaking water bath at 37 ^{o}C and 200 rpm, cultures were centrifuged, cell-free medium was diluted 30x with deionized water and absorption spectra were recorded. Controls containing PBL/glucose and 5.0 mM histidine were incubated under the same conditions. Line 1, control; line 2, incubated with *E. coli*.
The experiment was repeated three times with similar outcome. The graph shows original record of a single experiment. The arrow points to a new peak at 273.5 nm.

When the PBS/glucose histidine mixture was incubated for the same time with *E. coli* at 4°C, no detectable change in the absorption spectrum of the medium was observed.
