# Supplementary material for: Effect of growth media on the MTT colorimetric assay in bacteria
Source: PLoS One. 2019 Aug 27;14(8):e0219713. doi: 10.1371/journal.pone.0219713 (PMC6711527; doi:10.1371/journal.pone.0219713)
Supplement: S6 Fig — Spent medium was prepared by incubating mid log E. coli cultures in PBS/glucose, containing the indicated histidine concentrations. After 12 hours of incubation at 37 oC on a shaking water bath, cultures were centrifuged and cell-free spent medium was aliquoted in a 96-well plate (100 μl/well). Medium not incubated with cells was used for comparison. The experiment was repeated three times, each sample in triplicate. Mean ± SD is presented. (PDF) [file pone.0219713.s006.pdf]

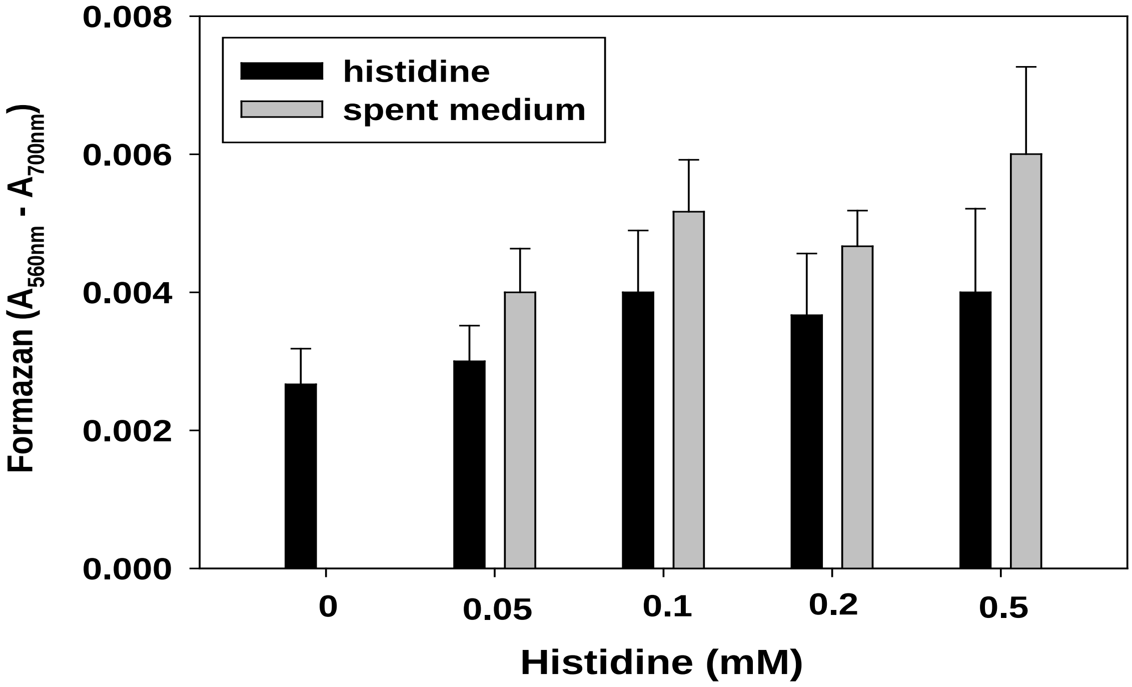


**S6 Fig. Effect of spent histidine medium on formazan formation in the absence of cells.** Spent medium was prepared by incubating mid log *E. coli* cultures in PBS/glucose, containing the indicated histidine concentrations. After 12 hours of incubation at 37 $^{o}$C on a shaking water bath, cultures were centrifuged and cell-free spent medium was aliquoted in a 96-well plate (100 μl/well). Medium not incubated with cells was used for comparison. The experiment was repeated three times, each sample in triplicate. Mean ± SD is presented.
