# Supplementary material for: Effect of growth media on the MTT colorimetric assay in bacteria
Source: PLoS One. 2019 Aug 27;14(8):e0219713. doi: 10.1371/journal.pone.0219713 (PMC6711527; doi:10.1371/journal.pone.0219713)
Supplement: S7 Fig — The cells of mid-log LB culture were thoroughly washed and resuspended to OD600 = 0.1 in PBS + glucose. Hundred μl aliquots were distributed to triplicate wells in 96-well plates, formamide, ammonium formate, ammonia, and histidine were added to a final concentration of 5.0 mM, and after 30 minutes of incubation, the MTT test was performed. The experiment was repeated two times, each sample run in triplicate. Mean ± SD is presented. (PDF) [file pone.0219713.s007.pdf]

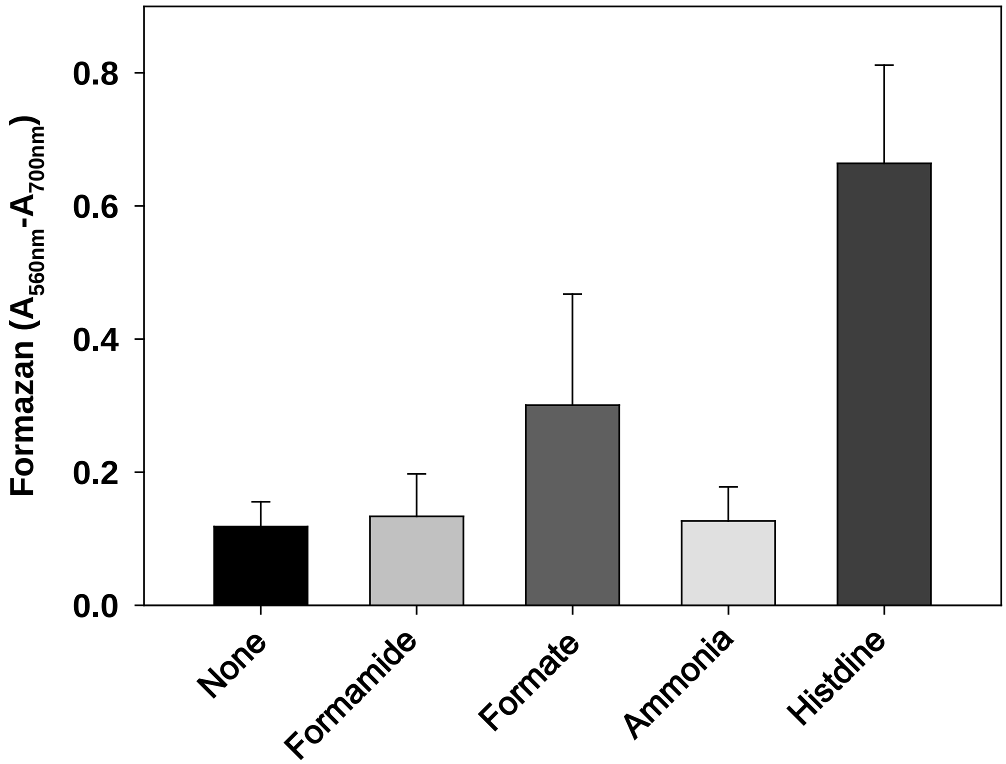

S7 Fig. Effect of histidine metabolic products on MTT reduction to formazan by *E. coli*. The cells of mid-log LB culture were thoroughly washed and resuspended to $OD_{600\ nm}$ = 0.1 in PBS + glucose. Hundred µl aliquots were distributed to triplicate wells in 96-well plates, formamide, ammonium formate, ammonia, and histidine were added to a final concentration of 5.0 mM, and after 30 minutes of incubation at 37 °C on a shaker, the MTT test was performed. The experiment was repeated two times, each sample run in triplicate. Mean ± SD is presented.
